# Is Percutaneous Endoscopic Gastrostomy A Solution for Aspiration Pneumonia, or Just An Illusion?

**DOI:** 10.5152/tjg.2025.24632

**Published:** 2025-03-28

**Authors:** Aycan Yüksel, Dorina Esendağlı, Polina Nezalzova, Gaye Ulubay

**Affiliations:** 1 Department of Respiratory Medicine, Başkent University Faculty of Medicine, Ankara, Türkiye

**Keywords:** Gastrostomy, pneumonia, aspiration

## Abstract

**Background/Aims:**

Aspiration pneumonia is a pulmonary infection that occurs when food, liquid, saliva, or vomit is aspirated into the lungs. Percutaneous enteral gastrostomy (PEG) prevents aspiration pneumonia while ensuring the patient receives adequate nutrition. The authors aimed to evaluate the incidence and risk factors of post-PEG aspiration pneumonia.

**Materials and Methods:**

Elderly patients who underwent PEG between January 2019 and December 2023 were included in this retrospective study. The incidence of aspiration pneumonia was compared between the periods before and after PEG. Mortality rates and risk factors of post-PEG aspiration pneumonia were investigated.

**Results:**

A total of 430 out of 534 patients who underwent PEG were included. The aspiration pneumonia rate was 30.1% (n = 133) before PEG and 21.9% (n = 94) after PEG (*P* = .003). The 1-year mortality rate of post-PEG aspiration pneumonia was 58.5% (n = 55). Age ≥ 80 years (OR: 3.11; 95% CI, 1.12-8.76), home residency (OR: 3.31; 95% CI, 0.99-10.8), discontinuation of primary caregiver who had been trained about PEG (OR: 5.8; 95% CI, 1.4-25.2), chronic lung disease (OR: 3.016; 95% CI, 1.17-7.77), history of recurrent aspiration pneumonia prior to PEG (OR: 3.401; 95% CI, 1.073-10.779), spending ≥20 hours of the day in supine position (OR: 6.512; 95% CI, 1.879-28.103), requiring PEG due to stroke (OR: 2.46; 95% CI, 1.062-5.69), and esophagus cancer (OR: 3.047; 95% CI, 1.174-8.88) were associated with post-PEG aspiration pneumonia.

**Conclusion:**

Percutaneous enteral gastrostomy reduces aspiration pneumonia in general, but there remain risks, particularly in patients with stroke or esophageal cancer. Supine position and discontinuation of primary caregiver who had been trained about PEG are major risk factors.

**Main Points**

- Percutaneous enteral gastrostomy reduces the incidence of aspiration pneumonia in general, but it does not completely eliminate the risk, even in patients without a history of prior aspiration pneumonia.
- Main risk factors for aspiration pneumonia in patients with percutaneous enteral gastrostomy are age (≥80 years), home residency, discontinuation of primary caregiver who had been educated and trained about percutaneous enteral gastrostomy, chronic lung disease, history of aspiration pneumonia prior to percutaneous enteral gastrostomy, stroke, esophageal cancer, and spending excessive time in the supine position.
- Proper education and training of caregivers, reducing the time spent in a supine position and ensuring that patients are placed in semi-recumbent or upright positions could lower the risk.

## Introduction

As the world’s population is getting older and older management of diseases and complications related to the aging of the body and organs has been the mainstay of health care providers. One of the most commonly seen age-related problems is swallowing dysfunction, which is sometimes difficult to diagnose and leads to aspiration of gastric content or microaspirations of oropharyngeal secretions into the lower respiratory tract, thus giving rise to inflammation and further infections, which are defined as aspiration pneumonitis or pneumonia respectively.^1^^,^^2^ The incidence of aspiration pneumonia is increasing and varies depending on the characteristics of patients and their age. Around 10% of hospitalized patients aged 80 or over with community-acquired pneumonia had an aspiration event, while in frail patients and residents of nursing homes this prevalance can be up to 10-fold.^3^^,^^4^ There are many factors that increase the risk for aspiration, such as poor oral hygiene, colonization of bacteria, malnutrition, alcohol consumption, usage of sedative drugs, seizures, central nervous system disorders including cerebrovascular diseases and also poor mobilization due to muscular diseases, etc.^1^ Since the morbidity and mortality are high in this group of patients, it is crucial to diagnose the presence of aspiration as soon as possible by performing a swallowing test.^5^

On the other hand, swallowing dysfunction is an important reason for poor and inadequate nutrition, which leads to weight loss. Thus, a percutaneous endoscopic gastrostomy (PEG), which enables feeding of patients directly into the gastric space, is one solution to not only prevent aspirations but also provide a way of feeding the patient.^6^ Percutaneous enteral gastrostomy has been shown to be superior to the nasogastric feeding tube regarding the improvement of nutritional status and reduction of complications.^7^ One study reported that PEG is a reliable, effective method associated with high satisfaction from 97% of patients who underwent PEG placement or their families.^8^ But long-term usage of PEG has itself been linked to aspiration pneumonia, and the procedure itself has its own complications with a high risk of morbidity and mortality.^9^^,^^10^ One study has even shown increased mortality in patients with dementia-related eating problems who had a PEG inserted compared to patients who did not.^11^

As PEG insertion and its benefits versus complications are still a topic of debate, it remains crucial to be very cautious while selecting the group of patients that might benefit the most from this procedure. This study aims to analyze the incidence of aspiration pneumonia before and after PEG insertion and thereby determine the extent to which PEG achieves its purpose, and to identify risk factors associated with morbidity and mortality in order to identify specific patient groups that may benefit most from this approach.

## Materials and Methods

### Study Design and Population

This single-center, retrospective study was conducted at Başkent University Ankara Hospital and included the elderly patients who underwent PEG between January 1, 2019, and December 31, 2023. All PEG procedures were performed by highly experienced gastroenterologists with a substantial track record in PEG placements. Patients who had undergone PEG at another center, those whose primary caregivers (such as family members, healthcare workers, or caregivers) were unable to receive training/education on PEG use, those who underwent other medical interventions (such as surgery, bronchoscopy, endoscopy) that could potentially cause aspiration pneumonia after PEG insertion, those who experienced conditions like gastric outlet obstruction, ileus, head trauma, or intoxication that could lead to aspiration pneumonia after PEG insertion, those diagnosed with malignancy after PEG placement, those with COVID-19 pneumonia, and those younger than 60 years were excluded. Data of enrolled patients were extracted from medical records, archives, and electronic patient database. This study was approved by Başkent University Institutional Review Board and Ethics Committee (project no.: KA24/293, date: August 20, 2024, approval number: E-94603339-604.01-370450) and was performed in accordance with the ethical standards laid down in the Declaration of Helsinki. Informed written consent was obtained from the patients or a legally authorized representative (such as a family member or legal guardian) prior to PEG insertion. Due to the retrospective design of the study, no additional written consent was required for their inclusion.

### Outcomes

The primary outcome was the annual incidence of aspiration pneumonia before and after PEG placement. As a secondary outcome, the risk factors for aspiration pneumonia following PEG placement were investigated. The mortality rate among those who developed aspiration pneumonia after PEG was also examined. The etiological conditions leading to the placement of PEG were identified. Additionally, the microorganisms cultured from lower respiratory tract samples (such as sputum, bronchial lavage fluid, and bronchoalveolar lavage fluid) of patients who developed aspiration pneumonia after PEG, along with thoracic tomography findings and laboratory results, were analyzed. The living conditions of the patients, including whether they resided at home or in a healthcare facility, as well as the identities of their primary caregivers (relatives, paid caregivers, or healthcare professionals), were assessed. Furthermore, it was examined whether these caregivers had received training regarding the care and use of PEG and whether those who had received such training continued providing care to the patients for up to 1 year.

### Statistical Analysis

The data were analyzed using SPSS version 22 (SPSS for Windows), (IBM SPSS Corp.; Armonk, NY, USA). The primary outcome, the comparison of pre-PEG and post-PEG aspiration pneumonia incidence, was conducted using the chi-square test. Based on the sample size calculation conducted via G-power 3.1, with an effect size of 0.3, an *α* of 0.05, and a power (1-*β*) of 0.95, the required minimum sample size was found to be n = 220. Risk factors associated with the development of post-PEG aspiration pneumonia were assessed using logistic regression analyses. For the secondary outcomes, comparing the hospitalization rates due to aspiration pneumonia before and after PEG, the chi-square test was similarly used. The duration of hospitalizations before and after PEG was compared with Student’s *t*-test. The frequency of microbiological agents, radiological findings, and mortality rates was evaluated with descriptive statistical analyses. Basic descriptive statistics (frequencies, proportions, means, and SD) were calculated, and 95% CIs were measured using standard statistical methods. Continuous variables following a normal distribution were presented as mean ± SD, while those not normally distributed were given as median and interquartile range (IQR, 25th-75th percentile). Categorical variables were presented as numbers and percentages. A *P*-value of <.05 was considered statistically significant.

## Results

**Figure 1.**
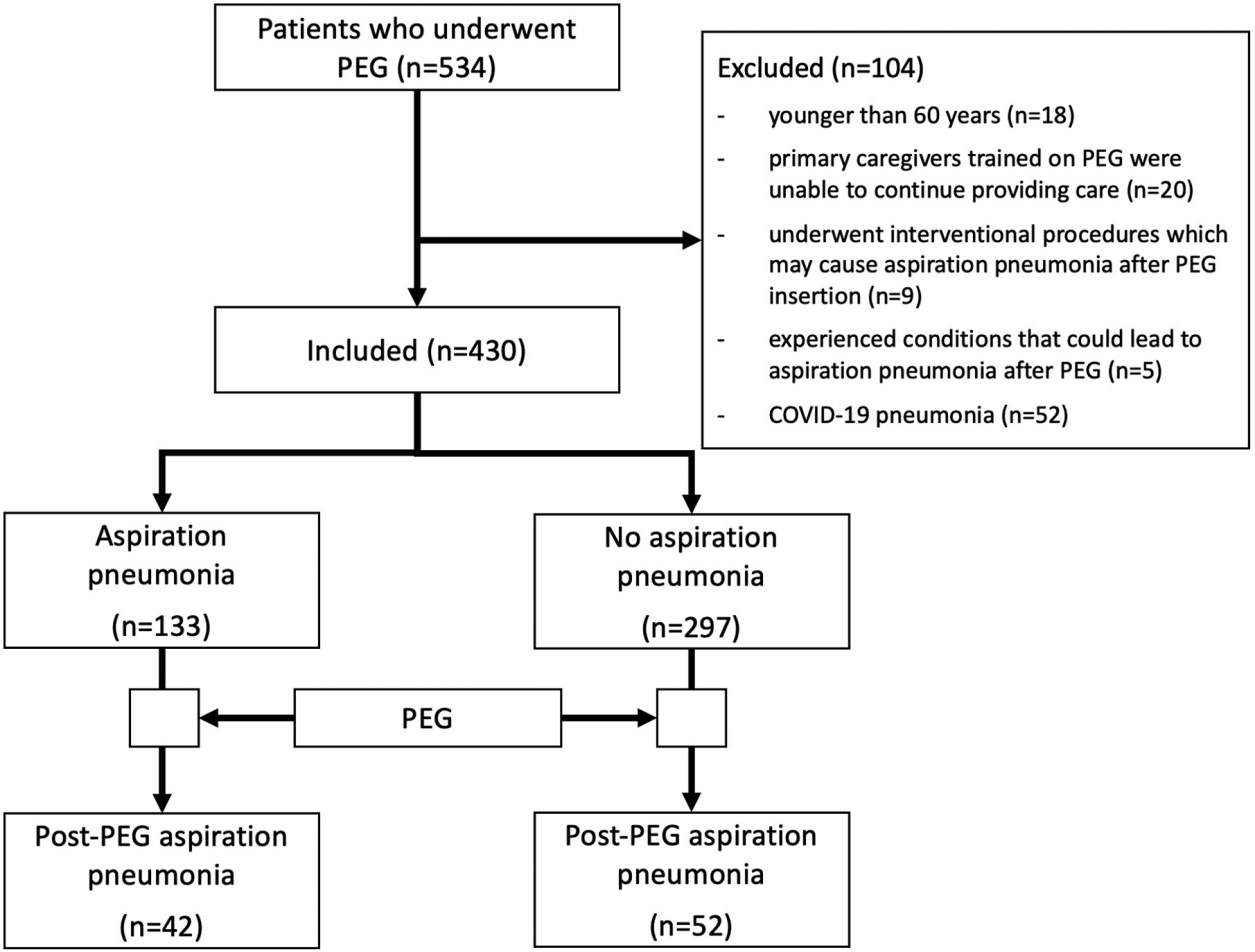
Flow diagram of the study.

**Figure 2.**
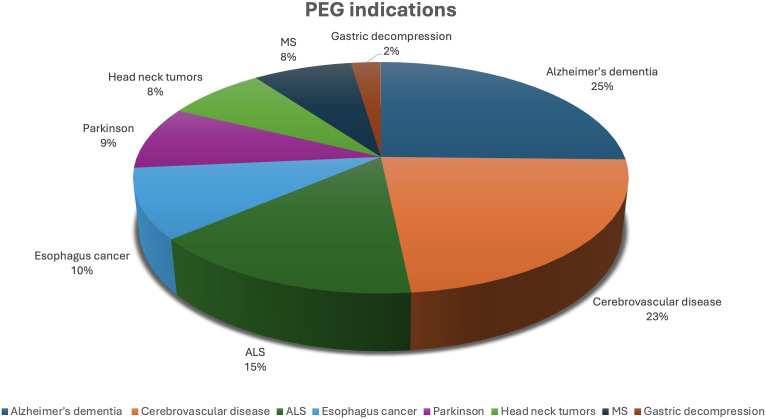
Prevalence of percutaneous endoscopic gastrostomy indications.

A total of 430 out of 534 patients who underwent PEG at our center during the study period were included in this study (Figure 1). The mean age of the patients was 77 ± 11 years, and 52% of the patients were female (Table 1). The baseline sociodemographic characteristics and comorbidities of the patients are shown in Table 1. Of the 430 patients, 313 (72.8%) resided in a nursing home or hospice facility, where their primary caregivers were nurses or other healthcare professionals. Of these 313 patients, 91 were residing in more specialized geriatric facilities affiliated with our academic tertiary hospital. In total, PEG tubes were placed in 430 patients, of whom 355 (82.6%) had non-malignant conditions and 75 (17.4%) had malignant disorders. The most prevalent indications for PEG were severe dementia due to Alzheimer’s disease (n = 109, 25.3%), cerebrovascular disease (n = 99, 23%), amyotrophic lateral sclerosis (n = 66, 15.3%), and esophageal cancer (n = 42, 9.8%) (Table 1, Figure 2). Intra-procedural endoscopic findings are summarized in Table 2, with the most common finding being a normal or mildly abnormal gastric mucosa in 70% of the patients. Gastroesophageal reflux was observed in 90 (20.9%) patients, and hiatal hernia in 87 (20.2%) patients (Table 2).

The most common complications after PEG were aspiration pneumonia (n = 94, 21.9%), local stoma site infection (n = 72, 16.7%), tube occlusion or dysfunction (n = 54, 12.6%), tube dislocation (n = 49, 11.4%), and peristomal leakage (n = 42, 9.8%). Major complications, such as buried bumper syndrome, peritonitis, and abdominal perforation, were infrequent (Table 3), and no episodes of major bleeding occurred.

**Figure 3.**
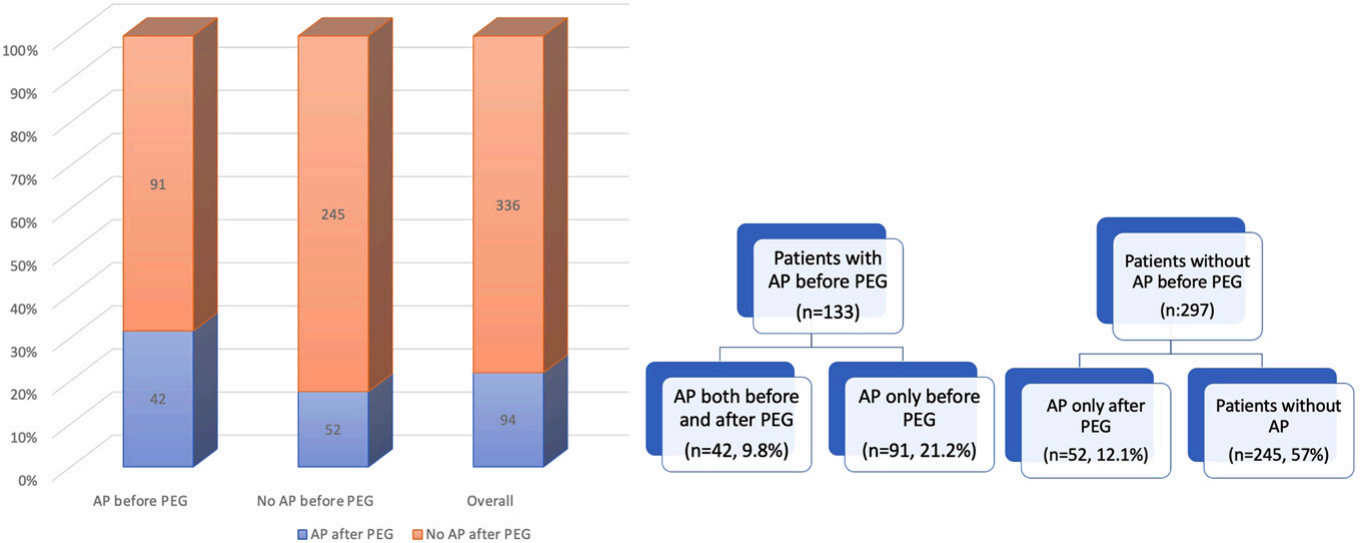
Comparison of post-PEG aspiration pneumonia incidence between patients with and without aspiration pneumonia prior to PEG.

The incidence of recurrent aspiration pneumonia was 30.1% prior to percutaneous PEG insertion, affecting 133 out of 430 patients. Following PEG placement, this rate significantly decreased to 21.9% (n = 94) (*P* = .003). Among the 133 patients who had developed aspiration pneumonia before PEG, 42 continued to experience recurrent aspiration pneumonia in the post-PEG period. The remaining 52 patients who developed aspiration pneumonia after PEG insertion were from the group of 297 patients who had not previously experienced aspiration pneumonia prior to the procedure (*P* = .002) (Figure 3). The incidence of post-PEG aspiration pneumonia did not differ significantly between patients with malignant (n = 19) and non-malignant (n = 75) indications for PEG placement (25.3% vs. 21.1%, *P* = .64; OR: 1.32; 95% CI [0.41-4.21]). The mean time to the onset of the first episode of aspiration pneumonia following PEG was 3.77 ± 0.61 months in patients who developed aspiration pneumonia post-procedure (Table 4). The 1-year mortality rate among those 94 patients who had aspiration pneumonia after PEG was 58.5% (n = 55).

The radiological features of the patients with aspiration pneumonia even after PEG are included in Table 4. Thoracic computed tomography (CT) of the study patients revealed consolidations, atelectasis, mucus secretions in bronchial lumen, and ground glass opacities in 73.4%, 48.9%, 29.8%, and 19.1% of the patients, respectively. The radiological lesions involved only dependent portions of the lungs, which are the posterior segments of the upper lobes and the basal segments of the lower lobes, in 66% of the patients, while 34% of the patients exhibited both dependent and nondependent lesions. Approximately 42.6% of the patients exhibited bilateral parenchymal infiltrates. The most frequently identified pathogenic microorganisms in lower respiratory tract cultures associated with aspiration pneumonia were *Klebsiella pneumoniae* in 26.6% (n = 25), anaerobic bacteria (including *Bacteroides* spp., *Fusobacterium* spp., and *Prevotella* spp.) in 23.4% (n = 23), and *Escherichia coli* in 20.2% (n = 19) of the patients (Table 4). Polymicrobial infections were observed in 67% (n = 65) of the patients. The results of serum infection biomarkers and total blood count are given in Table 4. The mean procalcitonin was 15.5 ± 5.8 μg/L, and the mean eosinophil count was 1155 ± 100 cells/μL.

Several factors were significantly associated with post-PEG aspiration pneumonia (Table 5). These factors included age older than 80 years (OR: 4.36; 95% CI, 1.63-9.45), home residency (OR: 3.51; 95% CI, 1.24-9.91), having chronic lung disease (OR: 6.51; 95% CI, 1.93-25.6), history of recurrent aspiration pneumonia prior to PEG (OR: 6.137; 95% CI, 1.54-26.123), spending ≥20 hours of the day in supine position (OR: 10.743; 95% CI, 2.892-40.005). Among primary caregivers, relatives (OR: 2.459; 95% CI, 1.062-5.69) and paid caregivers other than health care professionals (OR: 2.66; 95% CI, 1.086-6.51) were associated with post-PEG aspiration pneumonia. Furthermore, the discontinuation of primary caregiver who had been educated and trained about PEG (OR: 10.32; 95% CI, 3.001-38.21) was significantly associated with aspiration pneumonia. Patients who underwent the PEG procedure due to stroke (OR: 4.21; 95% CI, 1.209-14.715) and esophagus cancer (OR: 3.113; 95% CI, 1.164-8.322) were more likely to experience aspiration pneumonia. Multivariate regression analysis revealed that the following variables were associated with post-PEG aspiration pneumonia: age older than 80 years (OR: 3.11; 95% CI, 1.12-8.76), home residency (OR: 3.31; 95% CI, 0.99-10.8), discontinuation of primary caregiver who had been educated and trained about PEG (OR: 5.8; 95% CI, 1.4-25.2), chronic lung disease (OR: 3.016; 95% CI, 1.17-7.77), history of recurrent aspiration pneumonia prior to PEG (OR: 3.401; 95% CI, 1.073-10.779), spending ≥20 hours of the day in supine position (OR: 6.512; 95% CI, 1.879-28.103), requiring PEG due to stroke (OR: 2.46; 95% CI, 1.062-5.69) and esophagus cancer (OR: 3.047; 95% CI, 1.174-8.88) (Table 5).

## Discussion

This study’s findings highlight that PEG placement is associated with a significant reduction in the overall incidence of recurrent aspiration pneumonia. However, it does not completely eliminate the risk, and some patients (especially those who have already had experienced aspiration pneumonia) remain vulnerable. For patients who already had recurrent aspiration pneumonia prior to PEG placement (n = 133), a significant proportion (about one-third) continued to experience the problem even after the procedure. This suggests that while PEG might help some patients, it does not eliminate the risk of aspiration pneumonia in all cases. This could be due to ongoing issues with gastroesophageal reflux, poor oral hygiene, or other underlying conditions that PEG does not address. In the group of 297 patients who had no prior history of aspiration pneumonia before PEG, 52 (17.5%) developed aspiration pneumonia after PEG insertion. Our results suggest that while PEG insertion is associated with a reduction in the overall incidence of recurrent aspiration pneumonia, there is still a risk for patients without prior history to develop it after the procedure. This could suggest that PEG placement might introduce new risks, such as aspiration of gastric contents, reflux, or improper care of the PEG tube leading to infection.

These findings highlight the need for careful patient selection before PEG insertion. Before proceeding with PEG, patients should be assessed for risk factors that may contribute to aspiration pneumonia. Understanding these risks helps to anticipate post-PEG complications. Several risk factors that are significantly associated with post-PEG aspiration pneumonia were revealed in the data. Patients over 80 years old have a significant risk for post-PEG aspiration pneumonia, and this aligns with general clinical knowledge that advanced age is a major risk factor for aspiration pneumonia, especially in PEG patients.^12^^,^^13^ Pre-existing pulmonary disease significantly increases the risk 3.1- to 6.2-fold, especially in elderly patients.^14^ Having chronic lung disease increases the risk of aspiration pneumonia 3-fold. Chronic obstructive pulmonary disease or other lung conditions could impair the patient’s ability to clear secretions, leading to aspiration and subsequent pneumonia. A review outlines that patients with a history of aspiration pneumonia are at higher risk of recurrence.^15^ A strong correlation between past episodes of aspiration pneumonia and future occurrences after PEG was demonstrated by the findings. A history of recurrent pneumonia indicates an ongoing risk, as these patients likely have predisposing conditions like dysphagia (difficulty swallowing) or impaired protective reflexes. In a previous review, supine positioning has been associated with a significantly higher risk, with ORs around 2-4.^14^ A very high risk for patients who spend long periods (≥20 hours) lying flat is underscored by the findings. The supine position increases the likelihood of aspiration due to gravity, and patients who are immobile may not be able to shift positions to protect their airways effectively.

Stroke as an indication for PEG has been reported as a risk factor significantly associated with aspiration pneumonia.^16^^,^^17^ It was found that patients who underwent PEG due to stroke had an OR of 4.21 (univariate) and 2.46 (multivariate), indicating a heightened risk for aspiration pneumonia. Stroke patients often suffer from dysphagia and impaired cough reflexes, both of which increase the risk of aspiration.^18^ Also, impaired swallowing (dysphagia), compromised cough reflex, and altered consciousness may increase the risk of aspiration. Our findings showed that PEG placement due to esophageal cancer increased the risk of aspiration pneumonia. Several studies have examined the risk of aspiration pneumonia following PEG placement in patients with esophageal cancer.^19^^,^^20^ A study on patients with malignant esophageal obstruction found a high incidence of aspiration pneumonia, particularly in those receiving enteral feeding, with no significant reduction in risk compared to other feeding methods, like nasogastric tubes.^20^ While PEG placement is intended to help with nutrition, it may not completely eliminate aspiration risks in patients with esophageal cancer. It could be explained by several factors: swallowing difficulty, impaired protective reflexes, and gastroesophageal reflux disease. Esophageal cancer often causes dysphagia, which is a well-established risk factor for aspiration pneumonia due to tumor growth, leading to difficulty in swallowing. This increases the risk of aspiration (food, liquid, or saliva entering the airway instead of the esophagus), which can cause aspiration pneumonia. The cancer or related treatments (such as surgery, chemotherapy, or radiation) may impair the normal protective reflexes of the airway, further increasing the risk of aspiration. Esophageal cancer patients are prone to reflux, and reflux has been identified as a risk factor for aspiration pneumonia in multiple studies.^16^ Esophageal cancer or PEG placement might exacerbate GERD, which could increase the risk of aspiration of stomach contents, leading to pneumonia. Studies have shown that PEG placement does not always prevent reflux of stomach contents into the esophagus, thus not entirely preventing aspiration pneumonia.^19^^,^^20^

Home residency, living at home as opposed to in a healthcare facility, increases the risk of post-PEG aspiration pneumonia. This may be due to fewer healthcare resources, less frequent medical supervision, or differences in care quality at home compared to hospitals or long-term care facilities. The impact of the primary caregiver on post-PEG aspiration pneumonia was examined, and it was found that those whose primary caregiver is a relative (partner, children, grandchildren, etc.) or a paid carer who was not a healthcare professional are at higher risk for aspiration pneumonia than those who had been cared for by a healthcare professional. This suggests that non-professional caregivers might lack the necessary medical skills to properly manage patients with PEG tubes, particularly in preventing aspiration. Most importantly, the discontinuation of an educated/trained caregiver shows a strong association with increased risk (OR: 10.32; 95% CI, 3.001-38.21), highlighting the critical importance of specialized caregiver training in PEG management. This suggests that the type of caregiver and the continuity of care provided by trained individuals are crucial in minimizing the risk of post-PEG aspiration pneumonia.

Patients with pre-existing aspiration pneumonia should be closely monitored post-PEG, as a significant percentage will continue to experience recurrent pneumonia. Additionally, for patients without prior aspiration, strategies to minimize post-PEG complications, such as proper positioning during feeding and managing reflux, should be implemented.

In this study, the focus was on understanding which subgroups of patients benefit most from PEG and which factors contribute to the development of post-PEG aspiration pneumonia in elderly individuals. The results may help refine indications for PEG placement and improve postoperative management strategies. Hence, the findings have several important implications for daily clinical practice when considering and managing patients for PEG placement. These can guide clinical decision-making, patient care strategies, and post-PEG management. The following implications are suggested: 1) Patient selection with careful assessment of risks and benefits: PEG placement should be carefully considered for each patient, particularly for those with stroke and esophagus cancer. While PEG can reduce the risk in some, a significant portion may continue to experience recurrent pneumonia. Clinicians should weigh the potential benefits against the risks, especially in patients who are already at high risk. 2) Close monitoring of high-risk patients: Patients with risk factors for aspiration pneumonia should be monitored closely post-PEG for ongoing symptoms. This involves regular assessments for respiratory complications and ensuring that the feeding process minimizes aspiration risks (e.g., proper positioning during feeding, training of primary caregivers regarding PEG). 3) Education and training for caregivers: Educating healthcare providers and caregivers on the correct management of PEG tubes is essential. This includes ensuring that feeds are delivered appropriately, the tube is maintained in good condition, and signs of aspiration or infection are promptly identified. Caregivers should also be trained in positioning the patient correctly during and after feeds to reduce the risk of aspiration. 4) Management of new-onset aspiration pneumonia post-PEG: The development of aspiration pneumonia in previously unaffected patients post-PEG suggests that clinicians must remain vigilant, even in those considered low-risk. Follow-up should include respiratory assessments and a review of feeding techniques. In cases where new-onset aspiration pneumonia occurs, clinicians may need to modify feeding regimens or consider alternative interventions (e.g., jejunal feeding to bypass the stomach and reduce reflux risk). 5) Consideration of alternatives to PEG: In patients who continue to aspirate after PEG or who are deemed at particularly high risk of post-PEG aspiration pneumonia (such as stroke, esophagus cancer, pre-existing lung disease, oldest old age, supine position most of the day), alternative interventions should be considered. Options such as nasojejunal tubes, jejunostomy, or anti-reflux procedures might be more appropriate in certain cases. 6) Informed consent and patient/caregiver communication: Setting realistic expectations is crucial. Patients and caregivers should be informed that while PEG placement may reduce the risk of aspiration pneumonia, it is not a guaranteed solution. Clear communication about the potential risks of ongoing or new-onset aspiration is essential for informed decision-making. In patients with advanced neurological or terminal conditions, PEG insertion may not significantly improve quality of life or reduce aspiration risks. These discussions should be part of the decision-making process.

A key strength of our study is the exclusion of patients whose caregivers had not received proper training regarding PEG management. However, the study’s limitations include its retrospective design and being conducted at a single center. To confirm the identified risk factors, future research should involve multicenter studies with prospective designs.

In conclusion, PEG placement reduces the incidence of aspiration pneumonia in general, but there remain risks, particularly in patients with chronic lung diseases, stroke, or esophageal cancer, and those spending excessive time in the supine position. Reducing the time spent in a supine position and ensuring that patients are placed in semi-recumbent or upright positions as much as possible could help mitigate this risk. Special attention should be given to elderly patients and those with risk factors. The main risk factors appear to be the caregivers’ lack of PEG training and stroke as the indication for PEG. Proper education and training of caregivers, especially when caring for PEG patients at home, are crucial to reducing the risk of aspiration pneumonia. By addressing these risk factors, healthcare providers can potentially reduce the incidence of aspiration pneumonia following PEG placement.

## Figures and Tables

**Table 1.** Baseline Sociodemographic and Clinical Characteristics of the Patients

Characteristics	Results (n = 430)
Age (mean ± SD)	77.5 ± 10.9
Gender (n, %) Women Men	223 (52%) 207 (48%)
Comorbidities (n, %) Hypertension Heart disease Diabetes Lung disease	264 (61.4%) 122 (28.4%) 113 (26.3%) 56 (13%)
Residency (n, %) House Nursing home or hospice	117 (27.2%) 313 (72.8%)
Primary caregiver (n, %) Relative(s) Paid caregiver (non-healthcare worker) Healthcare worker or nurse	22 (5.1%) 95 (22.1%) 313 (72.8%)
Indication for PEG (n, %) Non-malignant disorders: Alzheimer’s dementia Stroke Amyotrophic lateral sclerosis Parkinson disease Multiple sclerosis Gastric decompression Malignant disorders: Esophagus cancer Head/neck tumors	355 (82.6%) 109 (25.3%) 99 (23%) 66 (15.3%) 38 (8.8%) 33 (7.7%) 10 (2.3%) 75 (17.4%) 42 (9.8%) 33 (7.7%)
Recurrent aspiration pneumonia before PEG (n, %)	133 (30.1%)

**Table 2.** Intra-Procedural Endoscopic Findings

Endoscopic Findings	Results (n = 430)
Normal or mildly abnormal gastric mucosa	301 (70%)
Mild esophagitis/gastritis	108 (25.1%)
Hiatal hernia	87 (20.2%)
Reflux changes (erythema, mild erosions)	90 (20.9%)
Significant ulcers	5 (1.2%)
Mass lesions or suspicious tumors (<5%)	3 (0.7%)
Severe esophagitis/strictures (<5%)	4 (0.9%)

**Table 3.** Percutaneous Endoscopic Gastrostomy Complications

Complications	Results (n = 430)
Aspiration pneumonia	94 (21.9%)
Local stoma site infection	72 (16.7%)
Tube occlusion or dysfunction	54 (12.6%)
Tube dislocation	49 (11.4%)
Peristomal leakage	42 (9.8%)
Pneumoperitoneum	21 (4.9%)
Minor bleeding	9 (2.1%)
Buried bumper syndrome	6 (1.4%)
Peritonitis	5 (1.2%)
Abdominal organ perforation	2 (0.5%)

**Table 4.** Clinical, Radiological, and Microbiological Characteristics of Patients with Post-PEG Aspiration Pneumonia

	Results (n = 94)
Aspiration pneumonia incidence after PEG	94/430 (21.9%)
Time to aspiration pneumonia after PEG procedure, months (mean ± SD)	3.77 ± 0.61
Mortality (n, %)	55 (58.5%)
Pathogen microorganisms (n, %) * Coinfection* * Klebsiella pneumoniae* * Anaerobic bacteria* * Escherichia coli* * Pseudomonas aeruginosa* * Acinetobacter baumannii* * Methicillin resistant staph aureus* * *Others	63 (67%) 25 (26.6%) 23 (23.4%) 19 (20.2%) 16 (17%) 10 (10.6%) 5 (5.3%) 4 (4.3%)
Thorax CT findings (n, %) * *Consolidations * *Atelectasis * *Mucus secretions in bronchial lumen * *Ground glass opacities * *Tree-in bud pattern * *Abscess	69 (73.4%) 46 (48.9%) 28 (29.8%) 18 (19.1%) 13 (13.8%) 1 (1%)
Distribution of radiological lesions on CT * *Only in dependent areas * *Dependent + nondependent areas * *Bilateral involvement	62 (66%) 32 (34%) 40 (42.6%)
C-reactive protein (mean ± SD)	117 ± 96
Procalcitonin (mean ± SD)	15.5 ± 5.8
Leukocyte count (/mm^3^)	13527 ± 1211
Neutrophil count (/mm^3^)	10231 ± 5718
Neutrophil percentage (%)	79.4 ± 13.5
Lymphocyte count (/mm^3^)	1225 ± 84
Lymphocyte percentage (%)	11.7 ± 8.7
Eosinophil count (/mm^3^)	1155 ± 100

CT, computed tomography; PEG, percutaneous endoscopic gastrostomy.

**Table 5.** Univariate and Multivariate Logistic Regression Analysis for the Predictors of Aspiration Pneumonia in Patients with PEG

Predictor	Univariate Analysis		Multivariate Analysis	
	OR (95% CI)	*P*	OR (95% CI)	*P*
Age (≥80 years)	4.36 (1.63-9.45)	.02	3.11 (1.12-8.76)	.041
Residency at home	3.51 (1.24-9.91)	.017	3.31 (0.99-10.8)	.037
Relative caregiver	2.459 (1.062-5.69)	.036	1.18 (0.608-2.307)	.619
Paid caregiver	2.66 (1.086-6.51)	.032	1.02 (0.99-1.008)	.447
Discontinuation of caregiver educated about PEG	10.32 (3-38.2)	<.001	5.8 (1.4-25.2)	.015
Chronic lung disease	6.51 (1.93-25.6)	.007	3.016 (1.17-7.77)	.022
Recurrent aspiration pneumonia prior to PEG	6.137 (1.54-26.123)	.015	3.401 (1.073-10.779)	.038
Indication for PEG Stroke Esophagus cancer	4.21 (1.209-14.715) 3.113 (1.164-8.322)	.024 .025	2.46 (1.062-5.69) 3.047 (1.174-8.88)	.036 .042
Supine position most of the day (≥20 hours)	10.743 (2.892-40.005)	<.001	6.512 (1.879-28.103)	.008

PEG, percutaneous endoscopic gastrostomy.

## Data Availability

The data that support the findings of this study are available from the corresponding author upon reasonable request.
